# Cardiac stroke volume in females and its correlation to blood volume and cardiac dimensions

**DOI:** 10.3389/fphys.2022.895805

**Published:** 2022-09-27

**Authors:** Janis Schierbauer, Sandra Ficher, Paul Zimmermann, Nadine B. Wachsmuth, Walter F. J. Schmidt

**Affiliations:** ^1^ Division of Exercise Physiology and Metabolism, University of Bayreuth, Bayreuth, Germany; ^2^ Department of Sports Medicine/Sports Physiology, University of Bayreuth, Bayreuth, Germany; ^3^ Department of Cardiology, Klinikum Bamberg, Bamberg, Germany

**Keywords:** cardiac output, oxygen uptake, echocardiogaphy, impedance cardiography, hemodynamics, hemoconcentration, plasma volume shifts, carbon monoxide-rebreathing

## Abstract

We aimed to continuously determine the stroke volume (SV) and blood volume (BV) during incremental exercise to evaluate the individual SV course and to correlate both variables across different exercise intensities. Twenty-six females with heterogeneous endurance capacities performed an incremental cycle ergometer test to continuously determine the oxygen uptake (V̇O_2_), cardiac output (Q̇) and changes in BV. Q̇ was determined by impedance cardiography and resting cardiac dimensions by 2D echocardiography. Hemoglobin mass and BV were determined using a carbon monoxide-rebreathing method. V̇O_2max_ ranged from 32 to 62 mL·kg^−1^·min^−1^. Q̇_max_ and SV_max_ ranged from 16.4 to 31.6 L·min^−1^ and 90–170 mL, respectively. The SV significantly increased from rest to 40% and from 40% to 80% V̇O_2max_. Changes in SV from rest to 40% V̇O_2max_ were negatively (r = −0.40, *p* = 0.05), between 40% and 80% positively correlated with BV (r = 0.45, *p* < 0.05). At each exercise intensity, the SV was significantly correlated with the BV and the cardiac dimensions, i.e., left ventricular muscle mass (LVMM) and end-diastolic diameter (LVEDD). The BV decreased by 280 ± 115 mL (5.7%, *p* = 0.001) until maximum exercise. We found no correlation between the changes in BV and the changes in SV between each exercise intensity. The hemoglobin concentration [Hb] increased by 0.8 ± 0.3 g·dL^−1^, the capillary oxygen saturation (ScO_2_) decreased by 4.0% (*p* < 0.001). As a result, the calculated arterial oxygen content significantly increased (18.5 ± 1.0 vs. 18.9 ± 1.0 mL·dL^−1^, *p* = 0.001). A 1 L higher BV at V̇O_2max_ was associated with a higher SV_max_ of 16.2 mL (r = 0.63, *p* < 0.001) and Q̇_max_ of 2.5 L·min^−1^ (r = 0.56, *p* < 0.01). In conclusion, the SV strongly correlates with the cardiac dimensions, which might be the result of adaptations to an increased volume load. The positive effect of a high BV on SV is particularly noticeable at high and severe intensity exercise. The theoretically expected reduction in V̇O_2max_ due to lower SV as a consequence of reduced BV is apparently compensated by the increased arterial oxygen content due to a higher [Hb].

## Introduction

It is generally accepted that the variation in maximal stroke volume (SV_max_) is mostly responsible for the range of maximum oxygen uptake (V̇O_2max_) values in healthy, trained and untrained men and women (Bassett and Howley, 2000; Levine, 2008; Lundby et al., 2017). Although it has been shown that endurance trained athletes possess higher absolute SV_max_ values for a given body dimension (Wiebe et al., 1998; Warburton et al., 1999a; Zhou et al., 2001), there is still an ongoing debate about the course of the SV during dynamic exercise. Specifically, it is a question whether the SV increases until termination of exercise in healthy individuals or at maximum intensity (i.e., 10–30 s before exhaustion) the SV is lower than the values observed at submaximal intensities due to a regulatory limit of the heart (González-Alonso, 2008; Warburton and Gledhill, 2008). However, these conclusions often come from studies that either compared resting to maximum values and/or further included only a single value during submaximal conditions. This circumstance is often due to the methodological difficulties in the continuous determination of cardiac output (Q̇). However, it is well known that the SV can demonstrate different individual courses (Vella and Robergs, 2005), which makes it necessary to include several measurement points and thus a continuous monitoring.

It has been repeatedly demonstrated that higher SVs are typically the result of larger cardiac dimensions, an enhanced venous return and cardiac preload. The latter is mostly due to a genetically predetermined and/or training-induced larger blood volume (BV) (Higginbotham et al., 1986; Ferguson et al., 2001; Rowland, 2009; Siebenmann et al., 2015). In this context, it must be noted that the BV substantially decreases during incremental exercise (Kawabata et al., 2004; Travers et al., 2020), thus possibly exerting a negative impact on the SV course during progressive exercise. At the same time, however, these volume shifts also have a beneficial impact on the oxygen transport capacity due to the increase in hemoglobin concentration as we were recently able to demonstrate (Schierbauer et al., 2021).

Basic structural cardiac properties including left ventricular (LV) hypertrophy as result of endurance training also contribute to the ability to continuously increase the SV throughout exercise especially in endurance trained athletes (Finkelhor et al., 1986; Levy et al., 1993; Hoogsteen et al., 2003). Since there is an interaction between the hemodynamic changes that occur during exercise and the cardiac dimensions, e.g., as seen in eccentric cardiac remodelling after chronic volume overload following endurance training (Hellsten and Nyberg, 2015; Vega et al., 2017), both cardiac dimensions and BV need to be investigated in addition to the aforementioned continuous SV monitoring to detect underlying mechanisms for different SV courses during dynamic exercise. To the best of our knowledge, this has not yet been done.

Therefore, the aims of this study were 1) to continuously evaluate the individual SV and BV course across different exercise intensities, 2) evaluate the correlation between cardiac dimensions and SV and BV at rest and during exercise, respectively and 3) to quantify exercise-induced BV shifts and estimate their influence on the SV and arterial oxygen content (CaO_2_) during incremental cycle exercise.

## Materials and methods

### Participants

Twenty-six healthy, nonsmoking females with heterogeneous endurance capacities and without history of cardiac disease were included in the study (see Table 1 for subject characteristics). The participants provided written consent after they were informed about the content of the study, the associated risks and the possibility to withdraw without indication of any reason. The study was conducted in conformity with the declaration of Helsinki and Good Clinical Practice and the study protocol was approved by the ethics committee of the University of Bayreuth in Germany (O 1305/1 – GB).

**TABLE 1:** Subject characteristics (*n* = 26).

	Mean ± SD	Min	Max	95% CI
Age (y)	27.5 ± 5.9	19	40	25.1–29.9
Height (cm)	167.7 ± 6.5	154	180	165–170
Body mass (kg)	60.1 ± 7.0	47.5	73.5	58.1–63.9
BSA (m^2^)	1.69 ± 0.1	1.43	1.88	1.64–1.74
BMI (kg·m^−2^)	21.6 ± 1.6	18.6	25.1	20.9–22.3
Lean body mass (kg)	47.4 ± 5.9	35.9	56.9	44.9–49.9
Fat mass (%)	22.2 ± 5.6	9.4	35.0	19.8–24.6
Ferritin (μg·L^−1^)	44 ± 24	16	105	34.2–54.0

The data are presented as the arithmetic mean ± standard deviation. Min, minimum; Max, maximum; CI, confidence interval; BSA, body surface area; BMI, body mass index.

### Study design

The participants performed an incremental cycle ergometer test during which the SV and V̇O_2_ were continuously measured. Simultaneously, the hemoglobin concentration for the calculation of BV and capillary O_2_ saturation (ScO_2_) were determined. Prior to the performance test, anthropometric measurements including body composition were conducted using a bioelectrical impedance analysis. A cubital venous blood sample was drawn to determine hematological variables and ferritin concentrations to exclude any iron deficiencies. The cardiac dimensions were determined by 2D echocardiography and the hemoglobin mass was measured twice on consecutive days and within 7 days after the ergometer test using a carbon monoxide-rebreathing method.

### Anthropometric measurements and analytical procedures

Prior to the exercise test, lean body mass and fat mass were measured twice consecutively and arithmetically averaged using a bioelectrical impedance analysis (InBody 770, InBody Co., Seoul, South Korea). The body surface area was calculated according to (Dubois and Dubois, 1916). Cubital venous blood samples (8 mL) were drawn after the participants rested for 15 min in a seated position. These heparinized blood samples were analyzed using a fully automated hematology system (Sysmex XN 1000-1-A, Sysmex, Norderstedt, Germany) for red blood cells including hemoglobin concentration ([Hb]) and hematocrit (Hct). In the serum, the ferritin and C-reactive protein (CRP) concentrations were determined by enzyme immunoassays [ferritin: LKFE1, CRP highly sensitive: LKCRP1 (ELISA & Immulite 1,000, Siemens Healthcare Diagnostics GmbH, Erlangen, Germany)].

### Incremental ergometer test

Maximum power output (P_max_) was determined using an incremental protocol on a cycle ergometer (Excalibur Sport, Lode, Groningen, Netherlands). After a 3-min warm-up phase of 50 W, the mechanical power output was increased by 50 W every 3 min (stepwise by 17, 17 and 16 W per minute) until subjective exhaustion was reached. The oxygen uptake (V̇O_2_) was determined via breath-by-breath technology (Metalyzer 3B, Cortex, Leipzig, Germany) and the maximum V̇O_2_ (V̇O_2max_) was calculated as the highest 30-s interval before exhaustion. In our analyses the values for V̇O_2_ were scaled to body mass as a standard reference and also to body mass to the power of −0.73 (Jensen et al., 2001). Capillary blood samples were taken from a hyperemized earlobe to quantify the lactate concentrations before exercise, every 3 min during exercise, immediately at exhaustion and 1, 3, 5 and 7 min after exhaustion (Biosen S-Line, EKF-Diagnostic, Barleben, Germany). Additional capillary blood samples were taken before exercise, every 3 min during exercise and immediately at exhaustion for the measurement of capillary oxygen saturation (ScO_2_, OSM III hemoximeter, Radiometer, Copenhagen, Denmark) and hemoglobin concentration ([Hb]) using a standardized and calibrated photometric analysis (HemoCue 201, Hemocue AB, Ängelholm, Sweden). Arterial oxygen content (CaO_2_) was calculated according to the following formula where 1.39 = Huefner number:

$${CaO}_{2\;}{(mL\cdot dL}^{-1})=\left[Hb\right]\;{(g\cdot dL}^{-1})\times {SpO}_{2}\;\left(\%\right)÷100\times 1.39\;{(mL\cdot g}^{-1})$$

### Calculation of cardiac output and arteriovenous oxygen difference during exercise

Stroke volume (SV) and cardiac output (Q̇) were measured continuously during exercise using a portable, battery powered and noninvasive cardiac monitoring device with signal morphology-based impedance cardiography (PhysioFlow Enduro, Manatec Biomedical, Paris, France). For a detailed description of the method see (Charloux et al., 2000). The values were continuously measured and averaged over 5-s intervals. For further analyses, four 5-s intervals were averaged for the calculation of the mean SV at the respective exercise intensities, i.e., 40% (SV_40%_), 60% (SV_60%_), 80% (SV_80%_) and 100% V̇O_2max_ (SV_100%_). Similarly, 5-s averages of SV data were aligned to absolute V̇O_2_ corresponding to 1.0, 1.5, 2.0, 2.5, 3.0 and 3.5 L·min^−1^. Based on the mean SV at a specific exercise intensity, we also calculated the changes in SV between exercise intensities, e.g., between SV_rest_ and SV_40%_ (∆SV_R-40%_) or SV_40%_ and SV_80%_ (∆SV_40–80%_). The highest SV (SV_max_) was calculated as the highest 20-s interval before exhaustion. Prior to the exercise, one investigator measured blood pressure using a professional blood pressure monitor (HBP-1300-E, Omron Healthcare Co., Ltd., Kyoto, Japan) with the participant seated and at rest on the cycle ergometer. Systolic and diastolic blood pressure were measured three times with the last two measures averaged for entry into the auto-calibration process of the PhysioFlow system. The arterio-venous oxygen difference (avDO_2_) was calculated according to the Fick principle, in which it represents the quotient of V̇O_2_ and Q̇. The PhysioFlow impedance cardiography was found to have an acceptable standard error of measurement of 3.96 at 70% of P_max_ (Gordon et al., 2018).

### Determination of hemoglobin mass and total blood volume

At least 2 h after the incremental test, when the plasma volumes had returned to preexercise values (Schmidt et al., 1990), the total hemoglobin mass (Hbmass), total blood (BV), plasma (PV) and erythrocyte (RCV) volumes were determined using a carbon monoxide (CO)-rebreathing method according to (Schmidt and Prommer, 2005; Gore et al., 2006; Prommer and Schmidt, 2007). In brief, an individual dose of CO (0.8–0.9 mL·kg^−1^, CO 3.7, Linde AG, Unterschleißheim, Germany) was administered and rebreathed along with 3 L of pure medical oxygen (Med. O_2_ UN 1072, Rießner-Gase GmbH, Lichtenfels, Germany) for 2 min. Capillary blood samples were taken before and 6 and 8 min after the administration of the CO dose. In the blood samples %HbCO was measured using an OSM III hemoximeter (Radiometer, Copenhagen, Denmark). The Hbmass was calculated based on the mean change in % HbCO before and after the CO was rebreathed. As part of the equation to calculate changes in BV during the exercise period, the capillary [Hb] was measured and converted to the venous conditions (Hütler et al., 2000; Patel et al., 2013). The BV was then calculated according to the following formula where 0.91 = cell factor at sea level (Fricke, 1965):

$$BV\;\left(mL\right)=Hbmass\;\left(g\right)\times 100÷\left[Hb\right]\;{(g\cdot dL}^{-1})\times 0.91^{-1}$$

The BV was calculated at rest (BV_rest_) and for different percentages of V̇O_2max_ (BV_40%_, BV_60%_, BV_80%_, BV_100%_). For the calculation of the submaximal BV the [Hb], which were determined at rest and every 3 min during exercise, were interpolated for the respective percentages of V̇O_2_, if necessary. We also calculated the exercise-induced changes in BV between the different exercise intensities, i.e., between BV_rest_ and BV_40%_ (∆BV_R-40%_), BV_40_ and BV_60%_ (∆BV_40–60%_), BV_60_ and BV_80%_ (∆BV_60–80%_), BV_80%_ and BV_100%_ (∆BV_80–100%_) and BV_rest_ and BV_100%_ (∆BV_R-100%_). Since the Hbmass does not change over short periods of time (Eastwood et al., 2008), the temporally offset determination of the [Hb] for the calculation of the BV is possible without compromising accuracy. For a detailed description and the accuracy of the method see (Schmidt and Prommer, 2005; Gore et al., 2006; Prommer and Schmidt, 2007). The typical error for the determination of Hbmass in our laboratory is 1.5%, which is in line with previous investigations (Hütler et al., 2000; Robertson et al., 2010), while the typical error for BV is 2.5%.

### Echocardiography

Transthoracic two-dimensional echocardiography for resting cardiac dimensions was performed by the same investigator with the participants remaining in a supine position using a cardiology ultrasound system (Philips EPIC 7, Phillips Medical Systems, Andover, MA, United States) with a 1.0–5.0 MHz sector array transducer (Philips S5-1, Phillips Medical Systems, Andover, MA, United States) according to the general recommendations (Lang et al., 2005; Evangelista et al., 2008). The systolic left ventricular ejection fraction (LV-EF) was estimated and calculated using the biplane Simpson rule, based on the apical four- and the apical two-chamber view. Two-dimensional linear dimensions for both ventricles and both atria were performed manually according to previous recommendations (Lang et al., 2005; Popescu et al., 2009). An estimation of the right ventricular systolic function using the tricuspid annular plane systolic excursion (TAPSE) was obtained in the apical four chamber view. Based on the 2D echocardiographic measurements, the left ventricular muscle mass (LVMM) and index (LVMM index), relative wall thickness (RWT) of the left ventricle and left atrial volume index (LAVI) were calculated with validated methods (Hashem et al., 2015; Lang et al., 2015). Additionally, each participant was evaluated for the prevalence of right and left heart valve regurgitation as part of the standard echocardiographic assessment with no participant demonstrating abnormalities.

### Statistical analysis

The data are presented as means and standard deviations. Statistical analysis was conducted using GraphPad Prism Version 8.0.2 (GraphPad Software, Inc., San Diego, United States) and IBM SPSS Statistics 26 (IBM, Armonk, United States). Testing for normality was performed using the Shapiro-Wilk test. Repeated measures one-way ANOVA or a mixed effects analysis followed by Turkey’s multiple comparisons test were performed to find significant differences between the exercise intensities. Pearson’s product moment and Spearman correlations as well as simple linear regression analyses were performed to assess the correlations and quantitative dependencies between two variables. The level of significance was set to *p* ≤ 0.05.

## Results

Performance, hematological and cardiac data showed large interindividual variability (see. Table 2). The V̇O_2max_ ranged between 32 and 62 mL·kg^−1^·min^−1^. The blood volume at rest (BV_rest_) and Hbmass ranged between 64 and 97 mL·kg^−1^ and 7.8 and 12.7 g·kg^−1^, respectively. LVEDV and LVEDD ranged between 50 and 150 mL and 32 and 52 mm, respectively. LVMM and LVMM index ranged between 65 and 187 g and 46 and 106 g·m^−2^.

**TABLE 2:** Performance, hemoglobin mass and cardiological data.

	Mean ± SD	Min	Max	95% CI
P_max_ (W)	259.3 ± 55.9	117	334	236–282
P_max_ (W·kg^−1^)	4.2 ± 0.8	2.3	5.5	3.9–4.5
V̇O_2max_ (mL·min^−1^)	2,992 ± 589	1,620	3,960	2,754–3,230
V̇O_2max_ (mL·min^−1^·kg^−0.73^)	149 ± 25	92	185	139–159
Hbmass (g)	597 ± 111	389	843	551–642
Hbmass (g·kg^−1^)	9.8 ± 1.2	7.8	12.7	9.3–10.3
RER_max_	1.22 ± 0.06	1.13	1.34	1.19–1.24
[Lac]_max_ (mmol·L^−1^)	12.1 ± 2.4	8.5	18.3	11.2–13.1
				
LVESV (mL)	35.3 ± 10.4	16	60	30.9–39.6
LVEDV (mL)	100.5 ± 27.8	50	150	88.8–112.2
LVEDD (mm)	41.0 ± 4.9	32	52	38.9–43.0
LVMM (g)	130 ± 35	65	187	115–145
LVMM index (g·m^−2^)	76.7 ± 17.5	46	106	69–84
PWd (mm)	9.6 ± 0.9	8	11	9.2–9.9
IVSd (mm)	9.9 ± 1.2	8	12	9.4–10.4

The data are presented as the means±standard deviations (SD). Min, minimum; Max, maximum; CI, confidence interval; P_max_, maximum power; V̇O_2max_, maximum oxygen uptake; V̇O_2max_ rel., relative maximum oxygen uptake; Hbmass, hemoglobin mass; Hbmass rel., relative hemoglobin mass; RER_max_, maximum respiratory exchange ratio; [Lac]_max_, maximum lactate concentrations; LVESV, left ventricular endsystolic volume; LVEDV, left ventricular enddiastolic volume; LVEDD, left ventricular enddiastolic diameter; LVMM, left ventricular muscle mass; PWd, left ventricular outflow tract diameter (mm); IVSd, interventricular septal thickness at end-diastole.

The BV significantly decreased until maximum exercise by 5.7% (280 ± 115 mL, *p* < 0.001); as a result, [Hb] significantly increased by 0.8 ± 0.3 g·dL^−1^. We found a moderate, yet not significant correlation between the BV_rest_ and the amount of fluid shifted until maximum exercise (∆BV_R-100%_, r = 0.38, *p* = 0.06). The ScO_2_ levels decreased by 4.0% (*p* < 0.001), while the CaO_2_ significantly increased from rest to maximum exercise (18.5 ± 1.0 vs. 18.9 ± 1.0 mL·dL^−1^, *p* = 0.001). The HR continuously increased until maximum exercise (184 ± 9.2 bpm). Q̇ increased in a linear fashion from 4.5 ± 1.4 L·min^−1^ at rest to 22.8 ± 3.6 L·min^−1^ at V̇O_2max_. The avDO_2_ significantly increased from 9.4 mL·dL^−1^ at 40% V̇O_2max_ to 13.1 mL·dL^−1^ at V̇O_2max_ (*p* < 0.001, see Table 3).

**TABLE 3:** Cardio-pulmonary data at rest and at different percentages of V̇O_2max_.

	Rest	40%	60%	80%	100%
V̇O_2_ (mL·min^−1^)	-	1,193 ± 233	1789 ± 350^*^	2,385 ± 466^*/#^	2,991 ± 589^*/#^
V̇O_2_ (mL·kg^−1^·min^−1^)	-	19.5 ± 3.2	29.3 ± 4.8^*/#^	39.1 ± 6.4^*/#^	49.0 ± 8.1^*/#^
Q̇ (L·min^−1^)	4.5 ± 1.4	12.7 ± 2.7^*^	16.1 ± 2.9^*/#^	19.3 ± 3.0^*/#^	22.8 ± 3.6^*/#^
Q̇ (mL·kg^−1^·min^−1^)	75 ± 21	211 ± 53^*^	269 ± 58^*/#^	319 ± 57^*/#^	377 ± 58^*/#^
SV (mL)	65 ± 17	113 ± 18^*^	116 ± 19	120 ± 18^#^	124 ± 20^#^
SV (mL·kg^−1^)	1.07 ± 0.24	1.87 ± 0.35^*^	1.93 ± 0.35	1.98 ± 0.33^#^	2.05 ± 0.32^#^
HR (1·min^−1^)	69 ± 12	112 ± 15^*^	139 ± 14^*/#^	161 ± 11^*/#^	184 ± 9^*/#^
avDO_2_ (mL·dL^−1^)	-	9.4 ± 2.8	11.1 ± 2.6^*/#^	12.4 ± 2.3^*/#^	13.1 ± 2.1^*/#^
∆BV_R-100%_ (mL)	-	−54 ± 56^*^	−173 ± 88^*/#^	−196 ± 105^*/#^	−280 ± 115^*/#^

The data are presented as the means ± standard deviations. V̇O_2_, oxygen uptake; Q̇, cardiac output; SV, stroke volume; HR, heart rate; avDO_2_, arteriovenous oxygen difference; ∆BV_R-100%_, changes in BV compared to resting conditions (^*^significant compared to previous intensity, ^#^significant compared to 40% V̇O_2max_, *p* < 0.05).

### Stroke volume response

**FIGURE 1:**
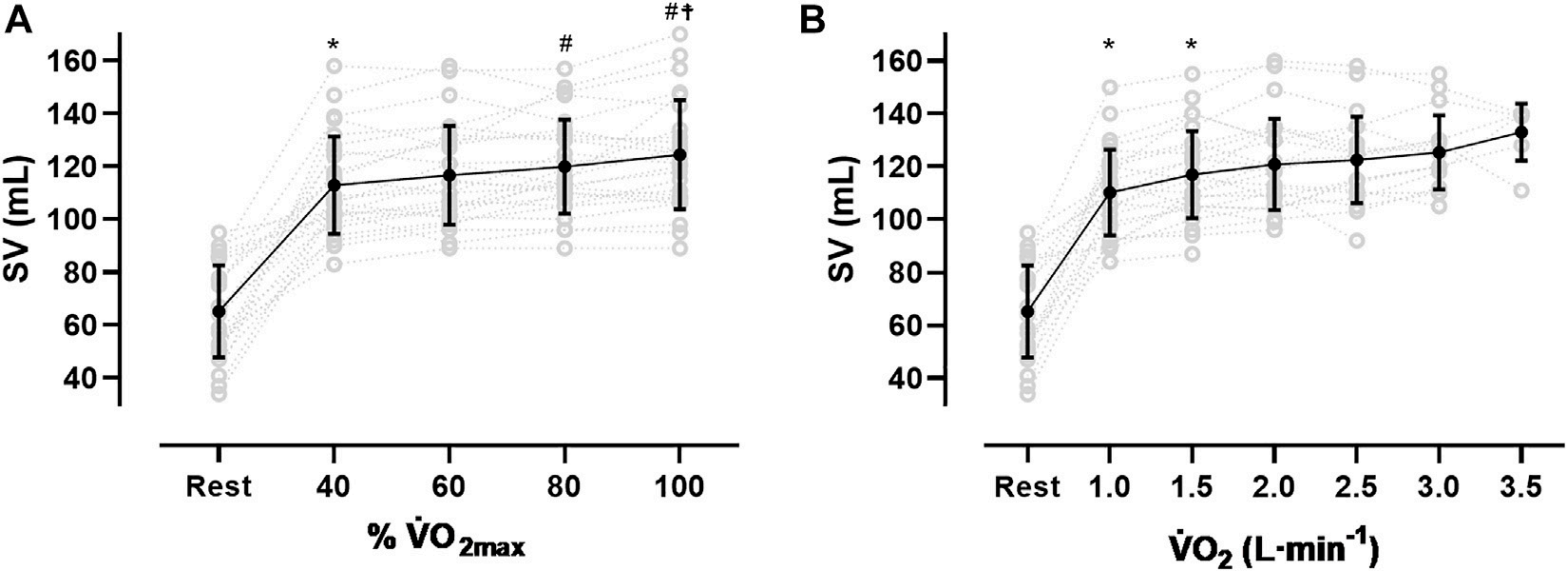
Stroke volume (SV) response from rest to V̇O_2max_ **(A)** and at the same absolute V̇O_2_ **(B)**, *sig. compared to previous condition, ^#^compared to 40% V̇O_2max_, ^☨^compared to 60% V̇O_2max_).

After an initial increase from rest to 40% V̇O_2max_ (65 ± 17 mL to 113 ± 18 mL, *p* < 0.001), mean SV significantly further increased from 40 to 80% V̇O_2max_ (120 ± 18 mL, *p* < 0.01) without significant change until V̇O_2max_ (124 ± 20 mL, see Figure 1A; Table 3). As Figure 1A shows, the SV response was highly individual including progressive increases (*n* = 8), plateaus with (*n* = 4) and without a secondary increase (*n* = 6) as well as plateaus with a drop (*n* = 4). Four participants showed a progressive increase until 80% V̇O_2max_ followed by a drop in SV. The ∆SV_R-40_ and ∆SV_40-80_ ranged from 8 to 98 and −7–20 mL, respectively.

The SV_max_ values ranged from 90 to 170 mL. Two participants reached their highest values at 40% V̇O_2max_, nine at 60%, five at 80% and ten at V̇O_2max_. The time point of reaching the SV_max_ was not significantly correlated to the relative V̇O_2max_ or the BV_rest_, even though for the latter a trend was observed (r = 0.37, *p* = 0.06). Subjects with a V̇O_2max_ ≥ 55 mL·kg^−1^·min^−1^ (*n* = 7) showed significantly higher SV values at all exercise intensities (*p* < 0.05) except for maximum exercise when compared to those with lower V̇O_2max_. When related to the same absolute oxygen uptake (Figure 1B), the magnitude of SV varied largely between participants specifically when absolute V̇O_2_ was low (e.g., 2000 mL·min^−1^: 117 ± 17.3 mL). At higher oxygen uptake values the scattering was considerably reduced (e.g., 3.5 L·min^−1^: 133 ± 10.7 mL).

### Correlation and regression analyses

#### SV vs. BV

SV_rest_ was significantly correlated to BV_rest_ (r = 0.70, *p* = 0.0001), however, the correlation between SV_40%_ and BV_40%_ was not significant. The correlations between SV_60%_ and BV_60%_ (r = 0.41, *p* < 0.05), SV_80%_ and BV_80%_ (r = 0.51, *p* < 0.01), SV_100%_ and BV_100%_ (r = 0.55, *p* < 0.01), as well as SV_max_ and BV_100%_ (r = 0.63, *p* < 0.001) were all significant. When these values were related to BSA, the correlations between the SV_rest_ and BV_rest_ (r = 0.59, *p* < 0.01) and SV_max_ and BV_100%_ (r = 0.42, *p* < 0.05) were still significant, whereas no correlations were found for the submaximal intensities. When the SV at different absolute V̇O_2_ values (0.5–3 L·min^−1^, see Figure 1B) were correlated with the BV_rest_, no significant correlations were found.

#### ∆SV vs. BV

**FIGURE 2:**
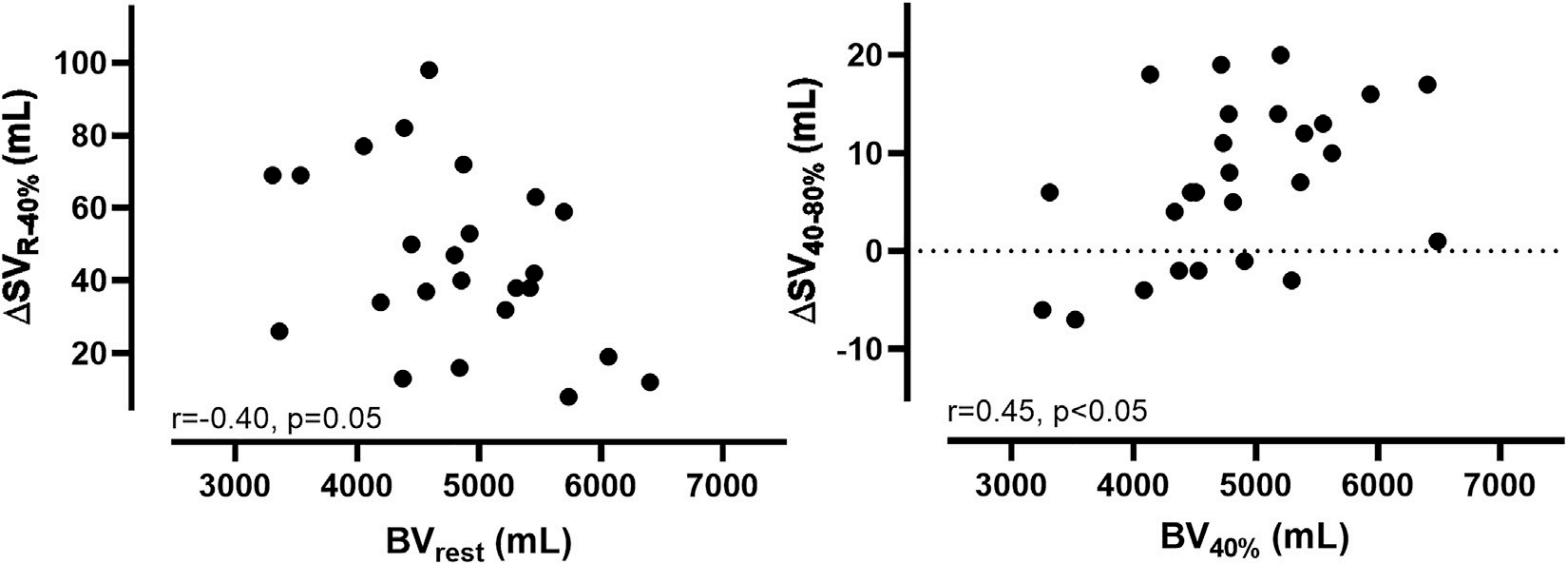
Correlations (*r*) and levels of significance (*p*) between the changes in stroke volume (∆SV) and blood volume (∆BV) from rest to 40% V̇O_2max_ and from 40% V̇O_2max_ to 80% V̇O_2max_.

While we found a negative correlation between the changes from SV_rest_ to 40% V̇O_2max_ (∆SV_R-40%_) and the absolute BV_rest_ (r = −0.40, *p* = 0.05), the correlation between ∆SV_40–80%_ and the absolute BV_40%_ was positively significant (r = 0.45, *p* < 0.05, Figure 2).

#### ∆SV vs. ∆BV

No correlation exists between the changes in SV and the exercise-induced BV shifts (e.g., ∆SV_40–60%_ vs. ∆BV_40–60%_) at the specific exercise intensities. The same also applies to the values related to BSA.

#### SV vs. cardiac dimensions

SV_rest_ was also significantly correlated to the LVMM (r = 0.52, *p* < 0.01) and LVEDD (r = 0.41, *p* < 0.05), respectively. When the LVMM and LVEDD were correlated to the SV at the other exercise intensities, the results were still significant (Table 4). We also found significant correlations between the SV_max_ and LVEDD (r = 0.70, *p* < 0.001) and LVMM (r = 0.68, *p* < 0.001, see Table 4), respectively.

**TABLE 4:** Correlations (*r*) and levels of significance (*p*) between the absolute and relative values of SV, BV and cardiac dimensions.

Variable A	Variable B	Absolute		Relative (·kg^−1^)		Relative (·kg^−0.73^/·m^−2^)	
		*r*	*p*	*r*	*p*	*r*	*p*
SV_max_ (mL)	LVEDD (mm)	0.70	<0.001	0.63	0.001	0.52	<0.01
	LVMM (g)	0.68	<0.001	0.56	<0.01	0.52	<0.01
LVEDV (mL)	BV_rest_ (mL)	0.69	<0.001	0.58	<0.01	0.59	<0.01
	BV_100%_ (mL)	0.65	<0.001	0.51	0.01	0.52	<0.01
LVEDD (mm)	BV_rest_ (mL)	0.60	<0.01	0.23	0.28	0.19	0.37
	BV_100%_ (mL)	0.62	<0.01	0.30	0.20	0.23	0.27
	SV_40%_	0.48	<0.05	0.63	0.001	0.44	<0.05
	SV_60%_	0.55	<0.01	0.67	<0.001	0.50	<0.05
	SV_80%_	0.61	<0.01	0.64	<0.001	0.47	<0.05
	SV_100%_	0.57	<0.01	0.54	<0.01	0.42	<0.05
LVMM (g)	BV_rest_ (mL)	0.78	<0.0001	0.61	<0.01	0.58	<0.01
	BV_100%_ (mL)	0.77	<0.0001	0.61	<0.01	0.58	<0.01
	SV_40%_	0.51	<0.05	0.52	<0.01	0.45	<0.05
	SV_60%_	0.57	<0.01	0.58	<0.01	0.52	<0.01
	SV_80%_	0.68	<0.001	0.61	<0.01	0.58	<0.01
	SV_100%_	0.51	0.01	0.47	<0.05	0.42	0.05

Data are related to body mass and body surface area. SV_40%_, stroke volume at 40% V̇O_2max_; SV_60%_, stroke volume at 60% V̇O_2max_; SV_80%_, stroke volume at 80% V̇O_2max_; SV_100%_, stroke volume at V̇O_2max_; SV_max_, maximum stroke volume; BV_rest_, blood volume at rest; BV_100%_, blood volume at V̇O_2max_; LVEDV, left ventricular end-diastolic volume; LVEDD, left ventricular end-diastolic diameter; LVMM, left ventricular muscle mass.

#### ∆SV vs. cardiac dimensions

The absolute and relative ∆SV_R-40%_ and ∆SV_40–80%_ were both not correlated to the LVMM and LVEDD, respectively. The correlations between ∆SV_60–80%_ or ∆SV_60–100%_ and the LVMM and LVEDD were also not significant.

#### Calculated dependencies between V̇O_2max_, Q̇ and BV

**FIGURE 3:**
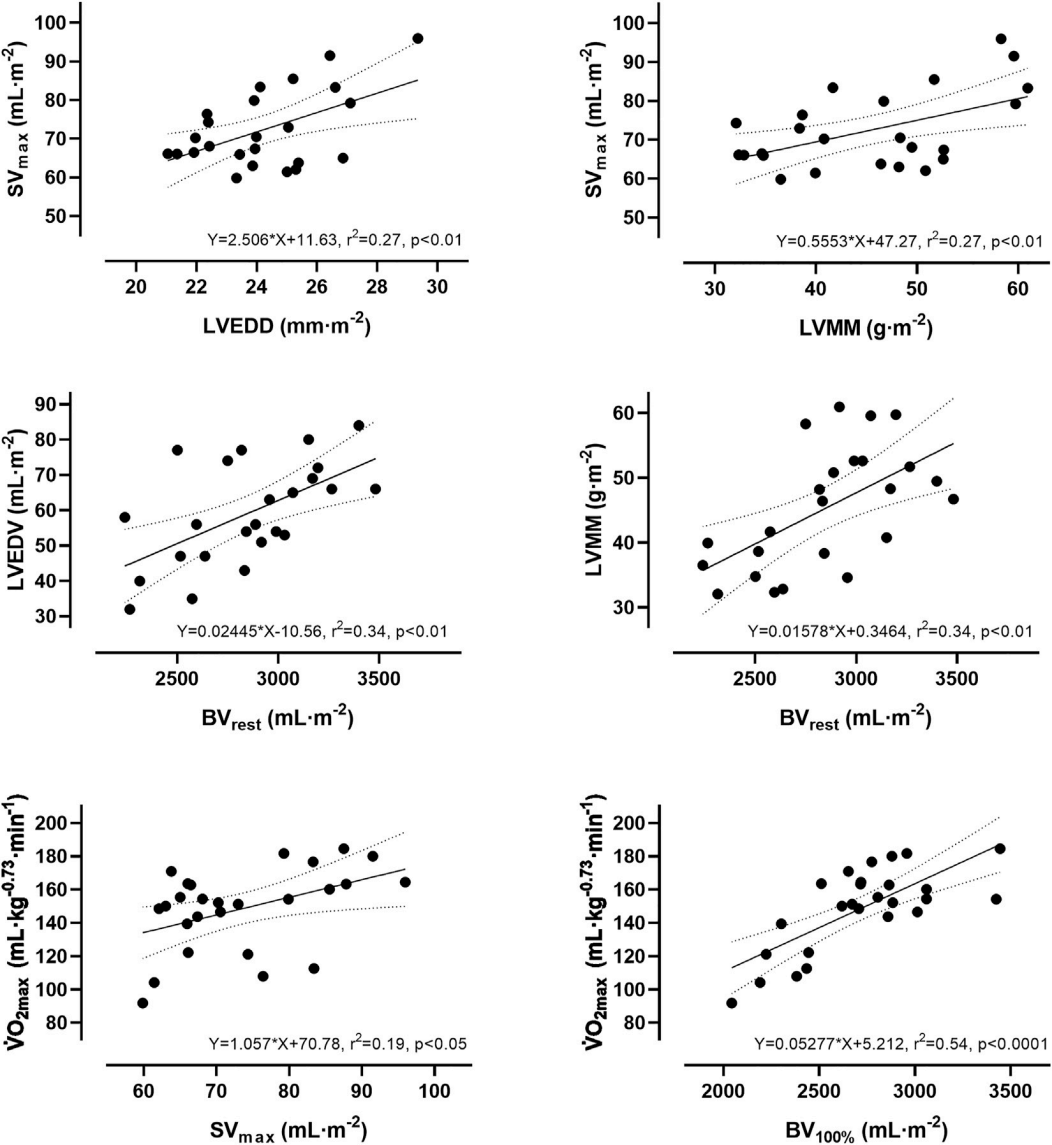
Simple linear regression analysis between absolute and relative values of stroke volume, blood volume, cardiac dimensions and maximum oxygen uptake.

The slope for an increase in Q̇ for every 1 L·min^−1^ increase in V̇O_2_ was ∼5.6 L·min^−1^ (y = 5.59x + 6.05). The slope of the respective regression line indicates that a 1 L higher Q̇_max_ was associated with a higher V̇O_2max_ of 104 mL·min^−1^. A 1 L higher BV_100%_ was associated with a higher V̇O_2max_ of 625 mL·min^−1^. According to the respective regression equation derived from the calculations in the Supplementary Material, a 1 L higher BV_100%_ was associated with a higher SV_max_ of 16.2 mL. Concerning the significant relationship between BV_100%_ and Q̇_max_, a 1 L higher BV_100%_ was associated with a higher Q̇_max_ of 2.5 L·min^−1^. Applying these cross-sectional data to the intra-individual changes in BV during exercise, the 5.7% reduction in BV found in this study would lead to a decrease in Q̇_max_ and V̇O_2max_ by 627 mL·min^−1^ and 156 mL·min^−1^, respectively. When the abovementioned variables were normalized to body mass and body surface area, the correlations were still significant (see Figure 3).

## Discussion

This is one of the few studies that continuously measured SV throughout incremental cycle exercise and, to the best of our knowledge, the first that included continuous BV measurements and correlated them to SV over the entire course of exercise. Our most important findings are that the mean SV response to dynamic cycle ergometer exercise does not seem to plateau in females with heterogeneous endurance capacities. The SV was significantly correlated to the BV at all exercise intensities, except BV_40%_. Individuals with the highest BV_rest_ showed the smallest changes in SV from rest to 40%, but the largest from submaximal intensities to V̇O_2max_. Basic resting cardiac dimensions were significantly correlated to the SV at all exercise intensities, but not to the changes in SV. The exercise-induced BV shifts were also not correlated to the changes in SV between the respective exercise intensities, however, they led to a higher oxygen transport capacity via an increase in [Hb].

### Stroke volume response

The mean values for SV_max_ in this study are similar to previous reports from moderately trained females (Wiebe et al., 1998; Wiebe et al., 1999; Ferguson et al., 2001), but smaller than in highly endurance trained females (Wang et al., 2012) and both endurance trained (Wiebe et al., 1998) and untrained males (Schierbauer et al., 2021). However, due to the methodology of continuous impedance cardiography and the resulting multiple measurement points, this study may provide further information. First, we identified all previously postulated SV responses to dynamic exercise (Vella and Robergs, 2005), which include progressive increases (*n* = 8), plateaus with (*n* = 4) and without a secondary increase (*n* = 6) as well as plateaus with a drop (*n* = 4). Additionally, we observed what was a progressive increase until 80% V̇O_2max_ followed by a slight drop in SV (*n* = 4, Figure 1A). Second, in this female population the SV response did not plateau at submaximal work rates but demonstrated a significant mean increase from 40% to 80% V̇O_2max_.

In the literature, there is still disagreement about the course of the SV response in healthy humans as they approach volitional exhaustion (González-Alonso, 2008; Warburton and Gledhill, 2008). However, it must be noted that the magnitude of the experimentally determined SV and thus Q̇ depends in general on both biological and methodological factors. Concerning the biological factors, previous studies have demonstrated that the SV may decrease at the end of an exhaustive test at least in untrained subjects (González-Alonso, 2008) which was explained by the regulatory limit of the heart. In contrast, it has also been demonstrated that especially in endurance-trained athletes, the SV does not plateau during exercise and that the additional increase was mainly attributed to enhanced diastolic function (Gledhill et al., 1994; Zhou et al., 2001). Even though we found a significant mean SV increase until 80% V̇O_2max_, the SV did in fact remain unchanged until exhaustion. Therefore, our results indicate that although no plateau was reached, the SV did not progressively increase until maximal effort, albeit no drop at exhaustion was discernible.

With regard to the methodological approach, it has been stated previously that the quantification of Q̇ also considerably depends on the applied method. For instance, in our recent study with males where we found a plateau in SV from submaximal to maximal power outputs (Schierbauer et al., 2021), we used an inert gas rebreathing method, whereas in this study an impedance cardiography was used. It is postulated that the first tends to underestimate Q̇ because of the recirculation of N_2_O which depends on the rebreathing time and exercise intensity (Jarvis et al., 2007), whereas the PhysioFlow is assumed to overestimate SV especially when thoracic blood volume decreases rapidly (Vieira et al., 2016; Cheung et al., 2020), although that does not seem to be a consistent finding (Charloux et al., 2000; Richard et al., 2001). In fact, studies reporting progressive increases throughout dynamic exercise mostly used an impedance cardiography method (Vieira et al., 2016). However, we found a similar slope for an increase in Q̇ per 1 L·min^−1^ increase in V̇O_2_ (∼5.6 L·min^−1^) using the PhysioFlow impedance cardiography as was reported in previous invasive investigations (Åstrand et al., 1964; Hermansen et al., 1970; Siebenmann et al., 2015). Therefore, we can assume that our data are valid and physiological conclusions can be drawn for the interpretation of the SV course, even though we did not perform multiple measurements to confirm consistent intra-individual SV profiles. The interpretation of the SV course and its comparison with the scientific literature must always include both biological and methodological factors, as we believe that a large proportion of the sometimes contradictory results may be due to different methodological approaches.

### Stroke volume and blood volume

In this study, we found significant correlations between the SV and BV at each of the exercise intensities, except for 40% V̇O_2max_ (see Supplementary Material). Furthermore, the correlations became even stronger with increasing exercise intensity. Our data also demonstrate that the smaller the BV_rest_, the higher the first increase in SV until 40% V̇O_2max_, as we found a trend towards a negative correlation between ∆SV_R-40%_ and BV_rest_. This is also supported by the inter-individual differences in the ∆SV_R-40%_ ranging from 8 to 98 mL indicating that in individuals with both a high BV_rest_ and SV_rest_ the hemodynamic output changes only to limited extent whereas individuals with a low BV_rest_ and SV_rest_ have to mobilize a higher percentage of their BV in order to meet their metabolic demand. In other words, it is likely that a larger BV_rest_ is associated with a larger central BV and therefore greater cardiac filling during diastole leading to a larger SV_rest_. In contrast, for a lower BV_rest_ and thus central BV there is more potential for an increase in cardiac filling and thus SV_rest_ due to peripheral volume shifts at the onset of exercise. As expected, the correlation is reversed in the further course of the exercise and becomes significant (∆SV_40–80%_ vs. BV_rest_, r = 0.45, *p* < 0.05). This might indicate that only individuals with an initially high BV are able to further increase SV at higher exercise intensities. Previous investigations have repeatedly demonstrated that a large BV is a prerequisite that allows for a larger SV during exercise due to an increase in central venous pressure and an elevated venous return (Levy et al., 1993; Krip et al., 1997; Martino et al., 2002; Bonne et al., 2014; Diaz-Canestro et al., 2021). This augments atrial and ventricular preload, which in turn enhances ventricular filling and results in an increased response of the Frank-Starling mechanism of the left ventricle (Ferguson et al., 2001).

At lower absolute oxygen uptake values the variability in SV was large which is most likely due to the heterogeneous endurance capacities of our participants. This would mean, that a given Q̇ could either be facilitated via a larger SV or HR. Therefore, a high BV would be less important for SV at submaximal intensities, which is supported by the nonexistent correlation between the BV and the SV at the same absolute submaximal V̇O_2_. However, it must be stated that at any intensity a high BV is generally beneficial for exercise performance as it allows for better thermoregulation (Sawka et al., 2000) or an improved lactate distribution (Wirtz et al., 2014). At high oxygen uptake values, however, the variability in SV becomes smaller, which might be due to the fact, that for achieving such high V̇O_2_ values, the SV becomes the limiting determinant, thus leading to converging high SV values (see Figure 1B).

In the context of the exercise-induced BV shifts, it remains uncertain if they might actually impair the SV course during dynamic exercise. While we found a linear decrease in BV, the SV in fact progressively increased until 80% V̇O_2max_. In addition, we found no correlation between the changes in SV (∆SV) and the exercise-induced BV shifts (∆BV) between any exercise intensity. It is therefore reasonable to assume that the volume shifts are fully compensated and even outmatched by an increased venous return (Berlin and Bakker, 2014). Nevertheless, the BV shifts may lead to SV_max_ and Q̇_max_ values that are possibly below the values, that would have been expected had no fluid been shifted. In this context it would be of great interest if volume matched fluid compensation during dynamic exercise in the extent of the individual volume shifts, e.g., via plasma or dextran infusion, would lead to a greater increase in SV and thus Q̇.

### Stroke volume and cardiac dimensions

It is generally accepted, that there is also a close relationship between the SV and the cardiac dimensions (Steding et al., 2010). We hereby confirm this relationship based on the significant correlation between the SV_rest_/SV_max_ and the LVMM and LVEDD. Additionally, we can also provide data for the submaximal intensities with the important limitation that only cardiac data at rest were available. Our data clearly demonstrate that throughout the exercise period, the SV is significantly correlated to the cardiac dimensions. This is in line with the general conviction, that larger cardiac dimensions are necessary to achieve higher SV values during dynamic exercise (Simone et al., 1992; Lauer et al., 1994; Daniels et al., 1995). In this study we also found significant correlations between the relative SV and cardiac dimensions indicating that when removing the influence of body size, the independent impact of other factors, e.g., training status, becomes apparent.

In contrast to the BV, we found no correlation between the cardiac dimensions and changes in SV (∆SV) throughout the exercise period. It can be concluded, that the sole consideration of the resting cardiac dimensions cannot explain the variation in SV and that additional factors, e.g., cardiac compliance or left-ventricular contractility also impact the behavior of the SV during dynamic exercise (Higginbotham et al., 1986). In general, our findings indicate that both the Frank-Starling mechanism and probably left ventricular function exert a substantial influence on the SV throughout exercise (Ferguson et al., 2001), even though we did not perform a strain imaging.

### Blood volume and cardiac dimensions

Since we and others have demonstrated that both the BV and cardiac dimensions determine the SV response to dynamic exercise, it is reasonable to assume that both parameters also influence each other. We were recently able to demonstrate that in males with heterogeneous endurance capacities there was a significant correlation between the BV and the resting cardiac dimensions, as was also found in this study. Higher BV typically result in an increased venous return and cardiac preload, thus serving for a potential stimulus for cardiac remodelling, e.g., after long-term endurance training (Arbab-Zadeh et al., 2014).

The connection between the BV and cardiac dimensions obtained from cross-sectional studies was also demonstrated when untrained subjects were matched to trained endurance athletes in terms of BV via dextran infusion, which led to an increase in SV in the untrained group. However, these values were still lower than those in the trained athletes (Hopper et al., 1988), which demonstrates that the BV alone cannot explain the differences in SV. Therefore, enhanced cardiac dimensions, cardiac contractility and/or other mechanisms, e.g., the ratio between hemodynamic active and inactive BV (Martino et al., 2002) may attribute to the SV response along with a larger BV.

### Fluid shifts and oxygen transport

Percentage changes in BV were calculated previously based on changes in [Hb] and Hct (Dill and Costill, 1974), but there are few studies available on absolute volume reductions during dynamic exercise. While we were recently able to demonstrate an 8% (∼550 mL) decrease in men with heterogeneous endurance capacities (Schierbauer et al., 2021), the female cohort in this study exhibited both a smaller relative and absolute volume reduction (∼6%, 280 mL). Other studies also demonstrated larger changes in BV in men when compared to the females in this study (Kawabata et al., 2004; Travers et al., 2020). These sex-specific differences are most likely due to the smaller active muscle mass during dynamic exercise in women (Myhre et al., 1985) whereas the fluid shifts are generally the result of a greater filtration rate caused by an increase in blood pressure, sweat loss and especially lactate accumulation and the breakdown of creatine phosphate within the muscle cell (Craig et al., 2008). The latter of which causes an increased osmotic gradient that, in turn, leads to an influx of water into the intracellular and interstitial space (Böning and Maassen, 1983; Maassen and Böning, 2008). Although we recognized a trend between ∆BV_R-100%_ and BV_rest_ (r = 0.38, *p* = 0.06), no correlation was found between the ∆BV_R-100%_ and the maximum lactate concentrations or V̇O_2max_, respectively. Therefore, it remains uncertain if endurance trained athletes shift more water into the intracellular space during incremental exercise.

In the context of the oxygen transport capacity, the volume shifts induced an increase in [Hb] by 0.8 g·dL^−1^ until maximum exercise that would theoretically lead to an increase in CaO_2_ by 1.1 mL·dL^−1^. However, due to the simultaneous decrease in ScO_2_ by ∼4%, the CaO_2_ increased by only 0.4 mL·dL^−1^. In this study population, the increase in [Hb] was lower and the decrease in ScO_2max_ was less pronounced when compared to males (Schierbauer et al., 2021) resulting in a similar increase in CaO_2max_ in both groups. It was previously demonstrated that a high Q̇_max_ may shorten the time for alveolar and capillary gas equilibration at the lungs which leads to an exercise-induced arterial hypoxemia that would reduce CaO_2_ (Goodrich et al., 2018). Since we found no correlation between Q̇_max_ and ScO_2max_, we hypothesize that the women’s Q̇_max_ values are too small to induce such a desaturation level. Our results clearly suggest that the BV shifts influence both oxygen transport capacity and Q̇. When comparing the calculated amount of oxygen transported in the arterial system either with or without the effects of the fluid shifts on [Hb] and Q̇_max_ the result is 4,314 mL·min^−1^ and 4,153 mL·min^−1^, respectively. This demonstrates that the fluid shifts more than compensate for the decrease in ScO_2_ and, if applicable, Q̇_max_. Like acute altitude effects, the hemoconcentration due to transient plasma volume shifts could, therefore, be interpreted as a physiological adjustment to maintain and in this case even improve oxygen transport capacity without compromising performance (Siebenmann et al., 2017).

### V̇O_2max_

The V̇O_2max_ is calculated on the basis of the Fick principle and therefore depends on several anatomical and physiological parameters (Lundby et al., 2017). We found the strongest correlation between the V̇O_2max_ and BV_max_ and Hbmass, respectively (see Supplementary Material). As derived from the respective regression equation, a 1 L higher BV was associated with a higher V̇O_2max_ by 625 mL·min^−1^. As we have already mentioned, the BV exerts a substantial influence on both the SV and the cardiac dimensions, whereas the Hbmass exerts a substantial influence on the oxygen transport capacity and avDO2_max_.

Even though the results from the cross-sectional study must be interpreted with caution, the possible influence of the exercise-induced BV shifts on the SV and the V̇O_2max_ can be estimated. Our results showed that a 1 L higher BV_100%_ was associated with a higher SV_max_ of approximately 16.2 mL, leading to a higher Q̇_max_ of approximately 2.5 L·min^−1^. As the BV significantly decreased from rest to maximal exercise by 280 mL (5.7%), we calculate a possible decrease in SV_max_ by 4.5 mL and in Q̇_max_ by 627 mL·min^−1^. As a consequence, without the compensation *via* the increase in CaO_2_ due to the change in [Hb], the V̇O_2max_ would also be reduced by 156 mL·min^−1^, which closely mirrors experimental data reporting a decrease by 125 mL·min^−1^ immediately after a blood volume reduction to a similar extent (Skattebo et al., 2021).

## Limitations

There are several limitations to this study. It is well known that the determination of Q̇ during exercise depends significantly on the applied method. The PhysioFlow impedance cardiography is assumed to be affected by movement under strenuous exercise, respiratory artefacts and possibly accumulation of fluid in the lungs which was associated with a high intersubject variance in previous studies (Warburton et al., 1999b; Peng et al., 2005; Siebenmann et al., 2015). This must be considered when interpreting the results of this study and comparing them with previous investigations. However, it is validated against the direct Fick method during exercise in healthy subjects (Richard et al., 2001) and its major advantage is in the continuous hemodynamic monitoring that allows a more comprehensive evaluation of the SV response. Additionally, the regression equation we calculated from the results in this study (Q̇ = 5.59*V̇O_2max_ + 6.05) is well in line with previous invasively measured data (Åstrand et al., 1964; Hermansen et al., 1970), which is a strong indication for the validity of the PhysioFlow, even though we did not perform multiple measurements to confirm consistent intra-individual SV profiles.

We have recruited a healthy population with heterogenous endurance capacities including sedentary, moderately and highly endurance trained participants to calculate correlations with an expected larger scattering of anatomical and physiological characteristics. For this reason, however, we did not have a sufficient number of participants in each of the aforementioned subpopulations and thus refrained from any statistical group analysis. We conducted correlation and linear regression analyses, thus we cannot confirm cause and effect. The quantitative dependencies that we drew from our analyses must therefore be interpreted with caution and are likely multifactorially driven. For the calculation of the possible influence of the BV changes on the SV course during dynamic exercise, we use data from the regression analyses, however, direct manipulations of BV must be applied to compare the effects of the volume shifts on hemodynamic mechanisms. Sweat loss estimates or direct measures of sweat loss should also be collected as they could impact the PV reductions during the incremental cycle ergometer exercise. To answer whether the increase in hemoconcentration and consequently the elevated CaO_2_ really are fully compensatory for the reductions in Q̇_max_ discussed here, additional data such as blood flow to working muscle, mean arterial pressure and vascular resistance should also be collected. Moreover, the present study cannot explain whether the increased CaO_2_ influences a regulatory feedback mechanism on the Q̇_max_ (Calbet, 2000).

## Conclusion

In 60% of our female participants with heterogeneous endurance capacities the SV does not plateau but progressively increases throughout incremental work until 80% V̇O_2max_. The BV does not seem to be relevant for the initial rise in SV but rather for increasing SV beyond submaximal exercise intensities. At all exercise intensities the SV was significantly correlated to the resting cardiac dimensions, which might be the result of adaptations to an increased volume load. The exercise-induced BV shifts may have a detrimental effect on the SV and V̇O_2max_, however, their negative effect on V̇O_2max_ is completely compensated for due to the increase in [Hb] and therefore arterial oxygen content.

## Data Availability

The raw data supporting the conclusion of this article will be made available by the authors, without undue reservation.
