# Supplementary figures and images for: Cardiac stroke volume in females and its correlation to blood volume and cardiac dimensions
Source: Front Physiol. 2022 Sep 27;13:895805. doi: 10.3389/fphys.2022.895805 (PMC9551173; doi:10.3389/fphys.2022.895805)

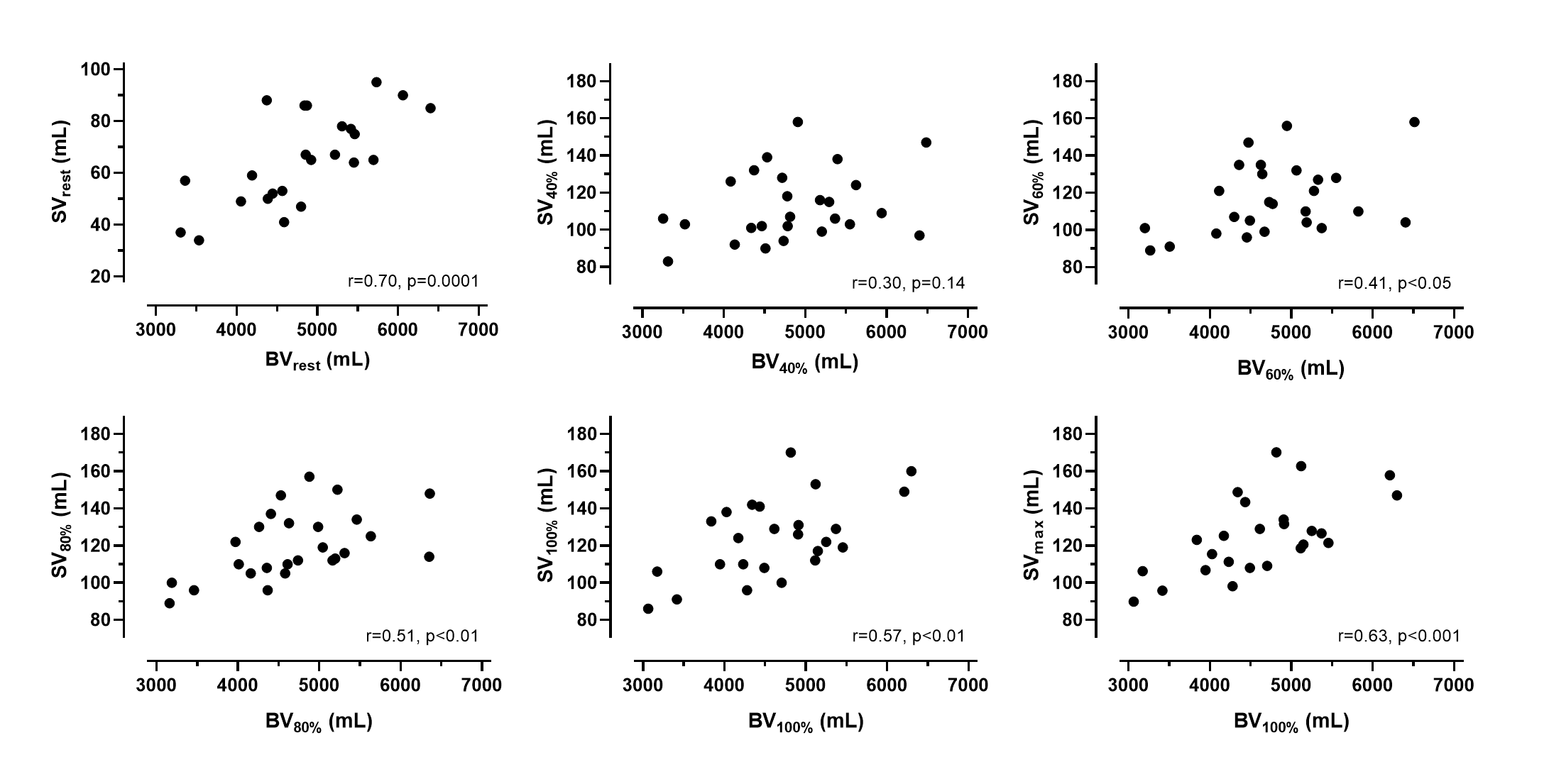

Supplement: Supplementary file 1 [file Image1.TIF]
