# Supplementary material for: Cardiac stroke volume in females and its correlation to blood volume and cardiac dimensions
Source: Front Physiol. 2022 Sep 27;13:895805. doi: 10.3389/fphys.2022.895805 (PMC9551173; doi:10.3389/fphys.2022.895805)
Supplement: Supplementary file 2 [file DataSheet1.DOCX]

**Supplemental Data**

**BV vs. cardiac dimensions:** The absolute BV_rest_ and BV_100%_ were both significantly correlated with the absolute LVEDD (r = 0.60, p < 0.01 and r = 0.62, p < 0.01) and LVMM (r = 0.78, p < 0.0001 and r = 0.77, p < 0.0001; Tab. 4). In addition, the absolute BV_rest_ was also significantly correlated with the absolute LVEDV (r = 0.69, p < 0.001). When related to BSA, these correlations were still significant.

∆**SV vs. V̇O_2max_:** When correlating the relative ∆SV_R-40%_ to the relative V̇O_2max_ (mL·kg^-0.73^·min^-1^) no correlation was found. When a sub analysis with participants with a V̇O_2max_ > 130 mL^-1^·kg^-0.73^·min^-1^ was performed the correlation was significant (r = 0.59, p = 0.01). With regard to the correlation between the relative ∆SV_40-80%_ and V̇O_2max_ (mL·kg^-0.73^·min^-1^) a positive trend was observed when all participants were included (p = 0.07).

**V̇O_2max_ vs. Q̇_max_:** We found significant correlations between the absolute (r = 0.64, p < 0.001) and relative (mL·kg^-0.73^·min^-1^, r = 0.43, p < 0.05, Tab. 5) values of V̇O_2max_ and Q̇_max_, respectively. The slope we found in this study for an increase in Q̇ for every 1 L·min^-1^ increase in V̇O_2_ was ~5.6 L·min^-1^ (y = 5.59x + 6.05). The correlations between the V̇O_2max_ and the other relevant variables are illustrated in Table 5.


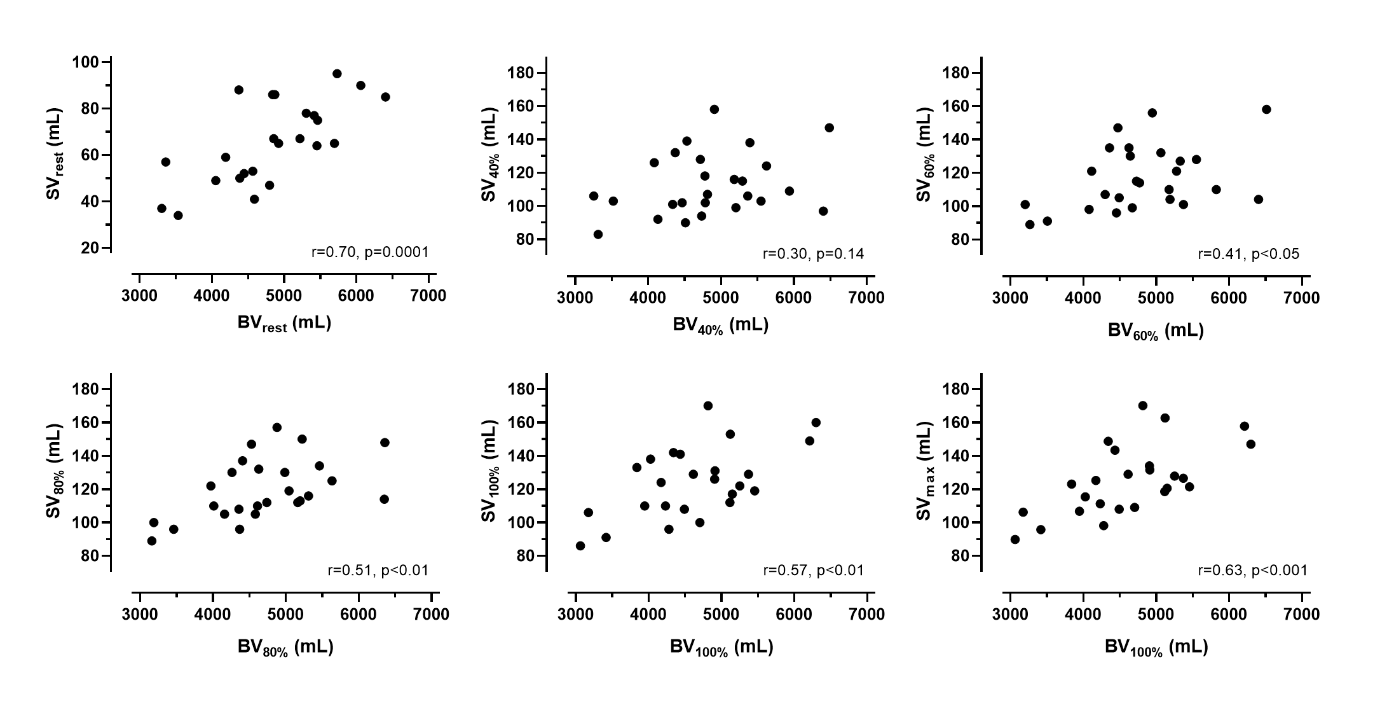


**Figure 4.** Correlations (r) and levels of significance (p) between the stroke volume (SV) and blood volume (BV) at different percentages of V̇O_2max_.

**Table 5.** Correlations (r) and levels of significance (p) between the absolute and relative values of V̇O_2max_, Q̇_max_ and relevant anatomical and physiological variables.

| **Variable A** | **Variable B** | **Absolute** | | **Relative** (·kg^-1^) | | **Relative** (·kg^-0.73^ /·m^-2^) | | |
| --- | --- | --- | --- | --- | --- | --- | --- | --- |
|  |  | *r* | *p* | *r* | *p* | | *r* | *p* |
| **V̇O_2max_ (L·min^-1^)** | Q̇_max_ (L·min**^-1^**) | 0.64 | <0.001 | 0.52 | <0.01 | | 0.43 | <0.05 |
|  | SV_max_ (mL) | 0.60 | 0.001 | 0.47 | <0.05 | | 0.47 | <0.05 |
|  | LVEDV (mL) | 0.59 | <0.01 | 0.42 | <0.05 | | 0.43 | <0.05 |
|  | LVEDD (mm) | 0.69 | <0.001 | 0.45 | <0.05 | | 0.38 | 0.07 |
|  | LVMM (g) | 0.76 | <0.0001 | 0.64 | <0.001 | | 0.61 | <0.01 |
|  | Hbmass (g) | 0.84 | <0.0001 | 0.74 | <0.0001 | | 0.78 | <0.0001 |
|  | BV_100%_ (mL) | 0.85 | <0.0001 | 0.78 | <0.0001 | | 0.78 | <0.0001 |
|  | avDO_2max_ (mL·dL**^-1^**) | 0.67 | <0.001 | 0.58 | <0.01 | | 0.51 | <0.01 |
|  | O_2_ EF (%) | 0.64 | <0.001 | 0.59 | 0.001 | | 0.63 | <0.001 |
|  | O_2_ TC (mL·min^-1^) | 0.65 | <0.001 | 0.50 | <0.01 | | 0.44 | <0.05 |
|  | ScO_2max_ (%) | -0.39 | <0.05 | -0.21 | 0.29 | | -0.28 | 0.16 |
| **Q̇_max_ (L·min^-1^)** | BV_100%_ (mL) | 0.56 | <0.01 | 0.39 | <0.05 | | 0.32 | 0.10 |
|  | SV_max_ (mL) | 0.94 | <0.0001 | 0.95 | <0.0001 | | 0.93 | <0.0001 |
|  | ScO_2max_ (%) | -0.18 | 0.36 | - | - | | - | - |
|  | HR_max_ (bpm) | <0.01 | 0.99 | - | - | | - | - |
